# Pulsatile Mass: Ruptured Common Femoral Artery Pseudoaneurysm with Active Extravasation

**DOI:** 10.7759/cureus.5380

**Published:** 2019-08-13

**Authors:** John O Patrick, Michael J Yoo, Neil P Larson, Rachel E Bridwell

**Affiliations:** 1 Emergency Medicine, Brooke Army Medical Center, Fort Sam Houston, USA

**Keywords:** pulsatile mass, ruptured, common femoral artery, pseudoaneurysm, active extravasation

## Abstract

Delayed rupture of a pseudoaneurysm represents an extremely rare and life-threatening complication of endovascular, radiographic, and cardiac procedures. We discuss a case of a 69-year-old man with delayed rupture of a known left common femoral artery pseudoaneurysm, highlighting the importance of rapid recognition, to include the use of point of care ultrasound, if available. Computed tomographic angiography allows for better anatomic characterization and aids in operative planning, which is the mainstay of treatment. However, surgical repair in ruptured pseudoaneurysms remains a high-risk procedure.

## Introduction

Pseudoaneurysms are a known complication of cardiac and vascular procedures [1]. While many of these pseudoaneurysms do not clinically deteriorate, rarer consequences include hemodynamic compromise, acute neurologic deficits, infection, expanding hematoma, and rupture [2]. Swift recognition and intervention can reduce morbidity and mortality. These complicated outcomes typically present in the days following a procedure and necessitate prompt surgical correction. We discuss an unusual presentation of a ruptured pseudoaneurysm in the setting of remote vascular intervention in a patient with extensive comorbidities.

## Case presentation

A 69-year-old man with a history of hypertension, hyperlipidemia, left-sided above the knee amputation secondary to severe peripheral artery disease with critical limb ischemia in 2012, tobacco abuse, and coronary artery disease on aspirin and clopidogrel presented to the emergency department (ED) complaining of left groin and scrotal swelling. The patient had a known history of a 4.9 cm left common femoral artery pseudoaneurysm, previously documented on a left lower extremity computed tomography (CT) angiogram in 2018. Based on chart review, this was likely a complication from an aortogram and right femoral and popliteal angioplasty accessed through his left common femoral artery in 2012. The patient had been followed by vascular surgery but previous attempts to repair his pseudoaneurysm were deferred secondary to the patient’s extensive comorbidities. The patient had been in his baseline condition without pain or swelling prior to this presentation. However, without any inciting factors, the patient stated that he felt a ‘pop,’ and over the next three hours noted acute left groin and scrotal swelling.

**Figure 1:**
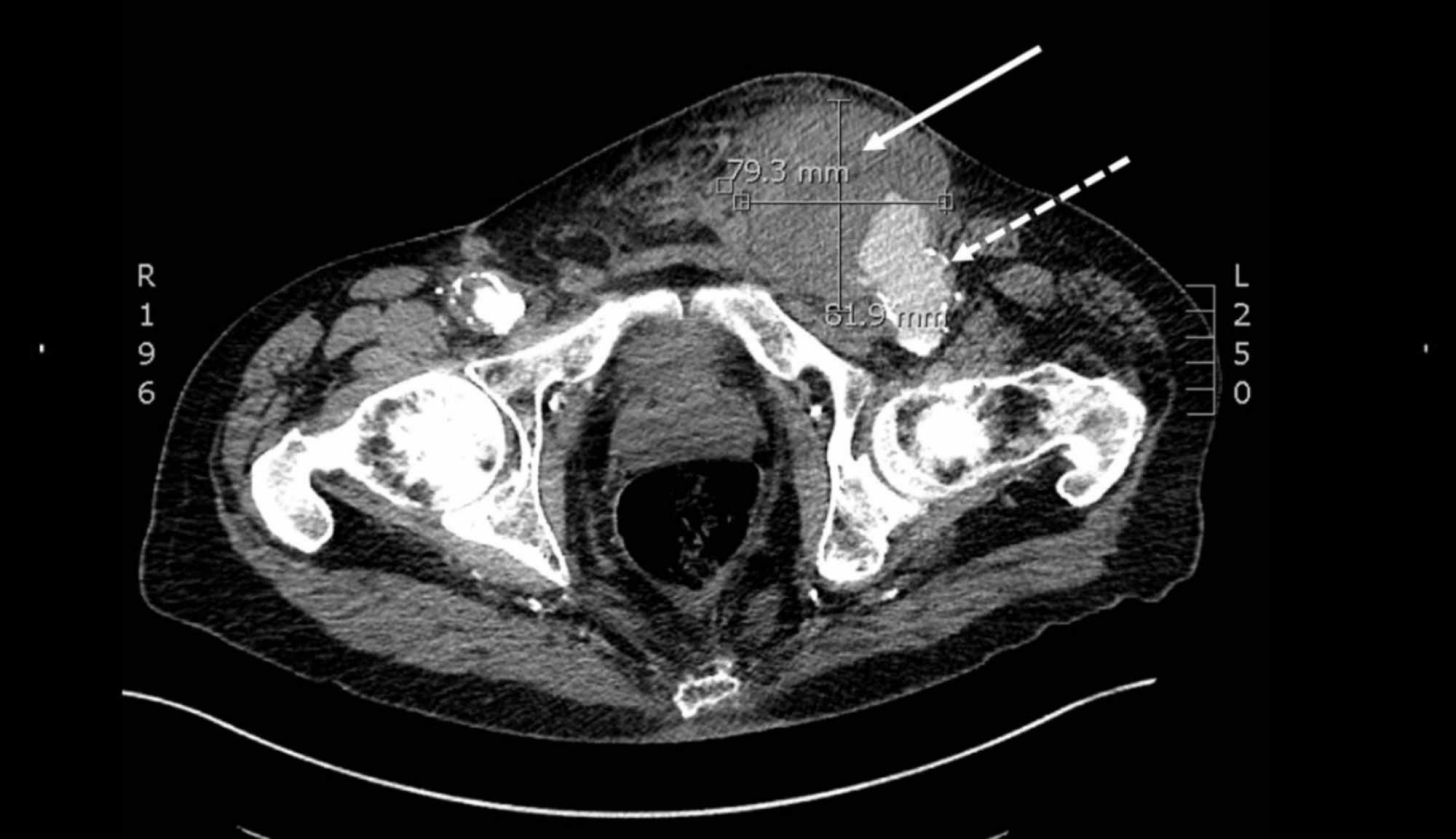
A transverse slice of a computed tomography pelvis angiogram demonstrating an 8.2 x 7.9 cm left common femoral artery (dashed white arrow) pseudoaneurysm (solid white arrow) with active extravasation

**Figure 2:**
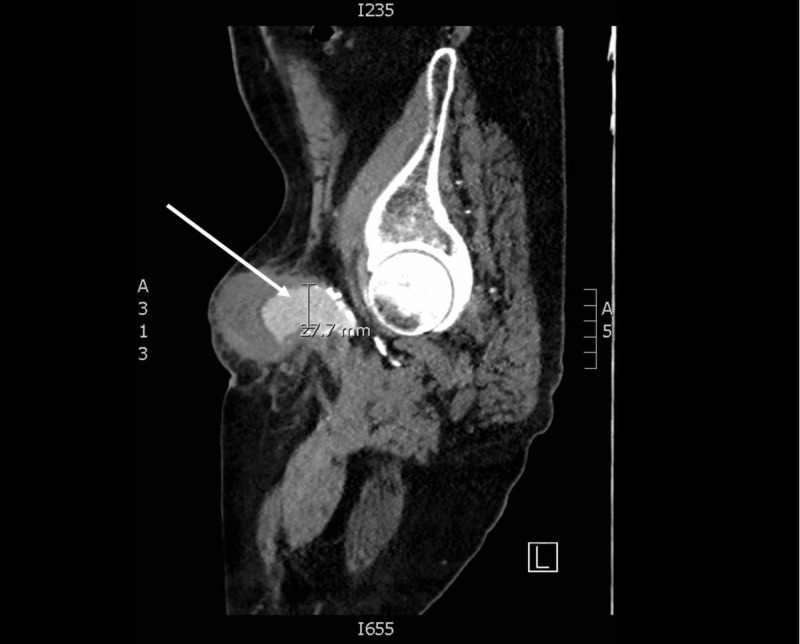
A sagittal slice of a computed tomography pelvis angiogram demonstrating 2.7 cm left common femoral artery at its widest diameter (solid white arrow)

On arrival, the patient’s vital signs included: blood pressure of 144/50 mmHg, heart rate of 79 beats per minute, temperature of 97.6⁰ Fahrenheit, and a pulse oximetry of 97% on room air. Physical exam was remarkable for a 15 cm, skin colored, pulsatile mass in his left inguinal region. However, the exam was negative for pain, overlying erythema, lesions, or external bleeding. The patient’s scrotum measured approximately 20 cm in diameter. A bedside ultrasound demonstrated approximately an 8 cm layering of new and old appearing clots, overlying a 2.3 cm diameter femoral artery. A subsequent CT pelvis angiogram was performed, revealing an 8.2 x 7.9 cm left common femoral artery pseudoaneurysm with active extravasation, extending down to the scrotum (Figures 1-2). 

In addition to the right hand 18-gauge angiocatheter and left hand 20-gauge angiocatheter established on arrival, a 7 French Rapid Infusion Catheter was placed in the right antecubital fossa. Serum studies resulted in a hemoglobin level at 13.1 g/dL; however, in anticipation of large volume bleeding, the patient was typed and crossed for four units of packed red blood cells. Based on the rapid interval increase in the patient’s pseudoaneurysm and now active extravasation, the patient was emergently taken to the operating room by general surgery. A perioperative chart review noted a pulsatile, ruptured, left common femoral artery pseudoaneurysm that was repaired with approximately 400 milliliters of blood loss. The patient's recovery was complicated by cardiac arrest secondary to ventricular fibrillation. One defibrillation shock was delivered with return of spontaneous circulation and without neurologic deficits. The patient declined additional procedures, including cardiac catheterization, and elected for hospice care.

## Discussion

Pseudoaneurysms are caused by a defect to the vessel wall that causes blood to accumulate in an adventitial or soft tissue sac; this is in contrast to true aneurysms in which the blood accumulates in a sac consisting of all layers of the blood vessel [3]. Pseudoaneurysms are a known complication of percutaneous arterial access for cardiac catheterization, peripheral artery disease, and therapeutic angiography, with an incidence ranging from 0.2% to 7.7% depending on type of procedure performed [4-5]. Risk factors for pseudoaneurysm development include access for intervention, rhythmologic procedures, size of sheath, and access through left groin, with the feared clinical course of wall expansion and rupture [2]. Management of pseudoaneurysms vary based on size, with asymptomatic ones less than 3 cm in diameter typically healing spontaneously and are observed as an outpatient with serial ultrasounds every two weeks [6].

Although rare, clinical features that should prompt ED clinicians for emergent intervention include hemodynamic compromise, acute neurologic deficits, concern for infection, and expanding hematoma [2]. Ruptured pseudoaneurysms are an even rarer phenomenon, with limited publications to date, and often occur within days of the initial inciting procedure [6]. In these cases of complicated pseudoaneurysm, open surgical repair remains the mainstay of treatment, which consist of pseudoaneurysm resection and arterial reconstruction [7-8]. These repairs, however, carry high complication rates in themselves couple with extensive comorbidities in a poor surgical candidate, with associated morbidity and mortality up to 71% and 2.1% respectively [1,3]. The most common complications include bleeding and infection, prompting acquisition of crossmatched blood in anticipation for peri-operative bleeding [5]. Nevertheless, in the setting of a ruptured pseudoaneurysm, emergent surgical consultation and intervention should be immediately pursued.

## Conclusions

In the setting of complicated pseudoaneurysms, rapid surgical intervention is indicated. Although delayed ruptures are rare, ruptured pseudoaneurysms are time-sensitive phenomena that carry high morbidity and mortality. Unfortunately, open repair remains an inherently high-risk procedure, especially in patients with extensive comorbidities.

## References

[1] Lumsden AB, Miller JM, Kosinski AS, Allen RC, Dodson TF, Salam AA, Smith RB (2019). A prospective evaluation of surgically treated groin complications following percutaneous cardiac procedures. Am Surg.

[2] Ahmad F, Turner SA, Torrie P, Gibson M (2008). Iatrogenic femoral artery pseudoaneurysms - a review of current methods of diagnosis and treatment. Clin Radiol.

[3] Stolt M, Braun-Dullaeus R, Herold J (2018). Do not underestimate the femoral pseudoaneurysm. Vasa.

[4] Katzenschlager R, Ugurluoglu A, Ahmadi A (1995). Incidence of pseudoaneurysm after diagnostic and therapeutic angiography. Radiology.

[5] Popovic B, Freysz L, Chometon F (2010). Femoral pseudoaneurysms and current cardiac catheterization: Evaluation of risk factors and treatment. Int J Cardiol.

[6] Kronzon I (1997). Diagnosis and treatment of iatrogenic femoral artery pseudoaneurysm: a review. J Am Soc Echocardiogr.

[7] Graham ANJ, Wilson CM, Hood JM, D'Sa AB (1992). Risk of rupture of postangiographic femoral false aneurysm. Br J Surg.

[8] Baldwin D, Mashbari H, Chow KL, Sarhan M (2019). Ruptured superficial femoral artery anastomotic pseudoaneurysm after 30 years. Case Rep Vasc Med.

